# YY1 is autoregulated through its own DNA-binding sites

**DOI:** 10.1186/1471-2199-10-85

**Published:** 2009-08-27

**Authors:** Jeong Do Kim, Sungryul Yu, Joomyeong Kim

**Affiliations:** 1 Department of Biological Sciences, Louisiana State University, Baton Rouge, LA, 70803, USA

## Abstract

**Background:**

The transcription factor Yin Yang 1 (YY1) is a ubiquitously expressed, multifunctional protein that controls a large number of genes and biological processes in vertebrates. As a general transcription factor, the proper levels of YY1 protein need to be maintained for the normal function of cells and organisms. However, the mechanism for the YY1 homeostasis is currently unknown.

**Results:**

The current study reports that the YY1 gene locus of all vertebrates contains a cluster of its own DNA-binding sites within the 1^st ^intron. The intact structure of these DNA-binding sites is absolutely necessary for transcriptional activity of the YY1 promoter. In an inducible cell line system that over-expresses an exogenous YY1 gene, the overall increased levels of YY1 protein caused a reduction in transcription levels of the endogenous YY1 gene. Reversion to the normal levels of YY1 protein restored the transcriptional levels of the endogenous YY1 to normal levels. This homeostatic response was also mediated through its cluster of YY1 binding sites.

**Conclusion:**

Taken together, the transcriptional level of YY1 is self-regulated through its internal DNA-binding sites. This study identifies YY1 as the first known autoregulating transcription factor in mammalian genomes.

## Background

The *Gli-Kruppel*-type transcription factor Yin Yang 1 (YY1) is a ubiquitously expressed, multifunctional protein that can function as an activator, repressor, or initiator binding protein depending on its promoter context, chromatin structure, and interacting proteins [1,2]. YY1 is also known to undergo various post-translational modifications, causing different outcomes on the functions of YY1. YY1 interacts directly or indirectly with many key proteins including the components of 1) RNA polymerase II transcription machineries, such as RNA polymerase II, TFIIB, TBP and TAFII55, 2) transcription factors, such as Sp1, c-myc, c-myb and CREB, and 3) histone-modifying complexes, such as p300, CBP, HATs, HDACs, PRMT1, and PhoRC and INO80 [3,4].

YY1 is involved in the transcriptional control of a large number of mammalian genes, approximately 10% of the total mammalian gene set [5]. Consequently, YY1 plays important roles in a number of biological processes, including cell cycle control, embryogenesis, viral infection, programmed cell death, oncogenesis, Polycomb Group (PcG) function and B-cell development [6]. Since it is a general transcription factor involved in so many pathways, the expression levels of YY1 must be tightly monitored for the survival of cells and organisms [4]. Accordingly, abnormal YY1 protein levels have been shown to cause defects in cell proliferation and differentiation, neural development, and the repression mediated by the PcG complex. Also, there are several types of the YY1-related diseases, such as viral infection and cancers, which are also linked to abnormal YY1 protein levels [3]. These observations clearly predict the presence of some regulatory mechanism(s) that are responsible for maintaining the appropriate transcriptional levels of YY1. Nevertheless, the mechanism of transcriptional regulation of the YY1 gene is largely unknown so far except for a few earlier observations on the promoter regions of the human and mouse YY1 gene [7,8].

In the current study, we have identified a cluster of multiple YY1 binding sites located in the first intron of the YY1 gene. We characterized this cluster of YY1 binding sites in terms of evolutionary conservation and transcriptional activity. We also tested the possibility that YY1 may be regulated through its own binding sites. For this test, we have established and used a Tet-On inducible system, in which we can control the cellular levels of YY1 protein. Our results demonstrated that changes in the cellular levels of YY1 protein affect the transcriptional levels of its own locus, YY1, most likely through its own binding sites. This autoregulation is regarded as a homeostatic response of the YY1 locus to maintain the constant cellular levels of YY1 protein.

## Results

### Identification of multiple YY1 binding sites in the 1^st ^intron of YY1

**Figure 1:**
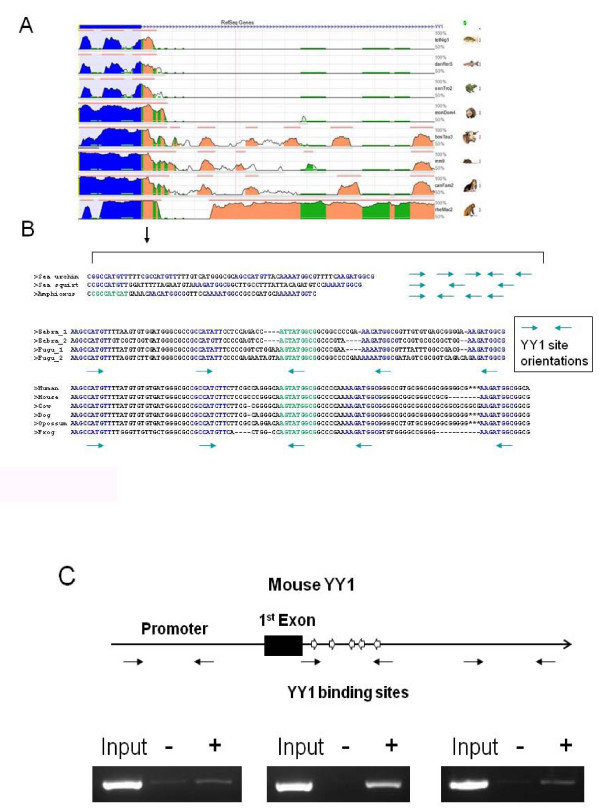
**Evolutionary conservation of the YY1 binding sites in the 1^st ^intron of YY1**. The ECR (Evolutionary Conserved Region) browser shows that the 1^st ^exon and the beginning region of the 1^st ^intron of YY1 are well conserved among eight species: tetraodon, zebrafish, frog, opossum, cow, mouse, dog, and rhesus macaque. Human YY1 was used as a reference sequence for this alignment. The height of the graph peaks indicate the level of nucleotide identity, while blue and salmon colors indicate exon and intron regions, respectively (**A**). The genomic sequences of the 1^st ^intron of YY1 from 11 different species were also manually aligned: one each from purple sea urchin, sea squirt and amphioxus, two from each of two fish, one from frog, and five from mammals. The YY1 binding sequences marked in blue and green indicate the perfectly and imperfectly-matched YY1 binding sequences, respectively. The arrows on the right side of the sequences or under the YY1 binding sequences indicate the orientation of individual YY1 binding sites (**B**). The genomic region surrounding the five YY1 binding sites located within the 1^st ^intron of mouse YY1. The YY1 binding sites are indicated by thick arrows with orientations, while the 1^st ^exon by a closed rectangle. The thin arrows underneath the genomic layout indicate the position and direction of primer sets used for the PCR amplification of ChIP DNAs. The amplified PCR products of each region are shown as the following order: Input (lane 1), the ChIP DNA with rabbit normal serum (lane 2), and the ChIP DNA with YY1 antibody (lane 3) (**C**).

We have identified an evolutionarily conserved region in the 1^st ^intron of YY1 via the ECR (Evolutionary Conserved Region) browser, a tool for visualizing sequence comparison of multiple vertebrate genomes [9]. As shown in Figure 1A, the genomic sequence of the 1^st ^exon and intron region of human YY1 was successfully aligned with those from eight different species including tetraodon, zebrafish, frog, opossum, cow, mouse, dog, and rhesus macaque. As expected, the first exon (marked in blue) showed high levels of sequence conservation. Interestingly, however, a similar level of conservation was also detected in the beginning of the 1^st ^intron region (marked in salmon). We further analyzed this small region, 100 bp in length, in more detail. As shown in Figure 1B, we manually aligned genomic sequences derived from 11 different vertebrates: one each from sea squirt, purple sea urchin and amphioxus, two from each of two fish, one from frog, and five from mammals. Multiple sequence alignment using ClustralW revealed 20 to 48% sequence identity between the urochordates, 71 to 82% sequence identity between the two fish, 80 to 98% sequence identity between the five mammals. Detailed inspection revealed that this small region is mainly filled with multiple YY1 binding sites. Most vertebrates contain 5 YY1 binding sites within this 100 bp region except for sea squirt (3 binding sites) and amphioxus (4 binding sites). Neither the sequence nor the length of the spacer regions between individual YY1 binding sites are conserved, which becomes more apparent in the urochordates' YY1 binding regions. The sequences of most of the YY1 binding sites are identical to the consensus sequences of the known YY1 binding motif, which display very high levels of affinity to YY1 protein [10]. However, one binding site, the 3^rd ^one of all the vertebrates except urochordates, shows a slight sequence variation from the known consensus (marked in green), but still conserved among the vertebrates. The orientation of the individual YY1 binding sites is also conserved: the first two YY1 binding sites are localized in one direction, while the remaining three sites are in the other direction. The opposite orientation of the two outside YY1 binding sites is particularly well conserved throughout all the vertebrates. We have also examined the 1^st ^intron of *pho*, a homolog of YY1 in *Drosophila *[11], but we did not find a similar genomic region with multiple Pho-binding sites. This indicates that this cluster of YY1 binding sites is unique to vertebrates. Chromatin immunoprecipitation (ChIP) assay was performed to confirm the *in vivo *binding of YY1 to its own binding sites located in the 1^st ^intron. Homogenized mouse brain tissues were cross-linked with formaldehyde and immuno-precipitated with anti-YY1 polyclonal antibodies (Figure 1C). As expected, the multiple YY1 binding sites showed consistent enrichment levels of the immuno-precipitated DNA with this ChIP experiment, whereas the two other control regions, upstream and downstream, did not show any detectible levels of the DNA enrichment. This confirms *in vivo *binding of YY1 to its own multiple binding sites in the 1^st ^intron. In sum, the 1^st ^intron of the YY1 gene locus of all vertebrates contains a cluster of its own DNA-binding sites.

### YY1 binding sites as a transcriptional activator for YY1 transcription

We performed promoter assays to determine potential roles of this cluster of YY1 binding sites in YY1 transcription. For this experiment, we first tried to clone and manipulate the 1-kb genomic region surrounding the 1^st ^exon of the mouse YY1, but we could not amplify and stably maintain this fragment in bacterial clones mainly due to the unusually high GC composition of this region, around 80%. Therefore, we used the homologous region derived from zebrafish. According to a series of initial pretests (data not shown), the promoter region of the zebrafish YY1 has reasonably high levels of promoter activity in several mouse cell lines, confirming the feasibility of the planned experiments as well as the evolutionary conservation of the promoter activity associated with this genomic region.

**Figure 2:**
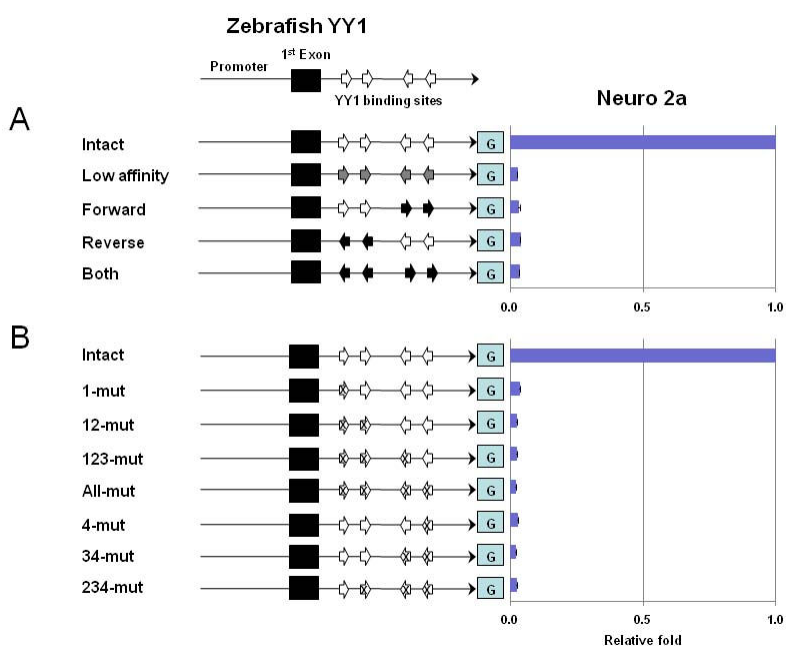
**The cluster of YY1 binding sites as a transcriptional activator**. The schematic diagram shows the promoter region of the zebrafish YY1 containing the 1^st ^exon and 4 perfect-matched YY1 binding sites (arrows). This promoter region was subcloned into the IRES-β-Geo promoterless vector, and subsequently modified to change the binding affinity and orientation of individual YY1 binding sites (**A**). Open arrows indicate the intact YY1 binding sites, while the gray arrows indicate the YY1 binding sites with lower affinity to the YY1 protein. All the YY1 binding sites in the Low affinity construct were modified from (A/C)GCCATnTT to AACCATnTT. The orientation of the YY1 binding sites marked by closed arrows is reversed with respect to the endogenous binding sites. The Forward construct has all binding sites in the forward direction; the Reverse construct has all in a reverse direction; and the Both construct contains both pairs in a reverse direction. The binding potential of these sites was also completely abolished through changing from GCCATnTT to ATTATnTT (**B**). The numbers in the name of each construct indicate the position of mutated YY1 binding site. The promoter activity of each construct was analyzed more than three times, and the averaged value is shown along with S.D. (Standard Deviation). The averaged value for each construct was further compared with that of the Control construct. These promoter assays were performed using two different cell lines, Neuro2A and NIH3T3. Only the result set from the Neuro2A cell line is shown in graphs since the result set derived from NIH3T3 showed almost identical patterns.

We have generated a series of 12 different constructs by modifying the 1.1-kb endogenous promoter region of the zebrafish YY1. The 1.1-kb region, covering the 1^st ^exon and the multiple YY1 binding sites, was first subcloned into a promoterless reporter system, IRES-β-Geo (Figure 2). Each of these constructs differs from the others in binding affinity, orientation and numbers of intact YY1 binding sites. This series of promoter assays excluded one particular YY1 binding site, the third, that imperfectly matches the YY1 consensus sequence (GCCATAnT instead of GCCATnTT). This difference is known to have much weaker binding affinity to YY1 protein [12]. To lower the DNA-binding affinity to the protein, the sequences of the 4 remaining YY1 binding sites were mutated from (A/C)GCCATnTT to AACCATnTT (Construct named as Low affinity). The orientation of YY1 binding sites was also reversed in an opposite direction (Constructs named as Forward, Reverse and Both in Figure 2A). We also completely abrogated the binding potential of individual YY1 binding sites through mutating the three critical bases within the core motif of YY1 from GCCATnTT to ATTATnTT (Constructs named as 1 mut, 12 mut, 123 mut, All mut, 4 mut, 34 mut and 234 mut in Figure 2B). These constructs were individually transfected into two different cell lines, Neuro2A and NIH3T3, along with an internal control luciferase vector (pGL3 control) to normalize the β-gal activity.

As shown in Figure 2A and 2B, the normalized transcriptional activity of each construct was compared to the activity of the control construct containing the original unchanged 1.1-kb genomic region of the zebrafish YY1. The promoter activities of all the mutant constructs showed a dramatic decrease compared to that of the control construct. The original activity was completely abolished by any change affecting the binding affinity or orientation of the intact YY1 binding sites (Figure 2A). This is also true for the second series of mutant constructs containing the different numbers of intact YY1 binding sites. Regardless how many and which positions of YY1 binding sites remain, a single change affecting any of the YY1 binding sites completely abolished the transcriptional activity of the YY1 promoter (Figure 2B). This indicates that all these YY1 binding sites are absolutely required for the transcriptional activity of the YY1 promoter. A similar pattern was also consistently observed in both Neuro2A and NIH3T3 cells. In sum, the YY1 binding sites function as a transcriptional activator for the YY1 promoter, and this role apparently requires the absolutely intact sequence structure of all the YY1 binding sites.

### Homeostatic control of the cellular levels of YY1 protein

The identification of the YY1 binding sites within the YY1 locus hinted at one intriguing possibility: YY1 may control its own transcription rate through its own binding sites. To test this possibility, we first set up an *in vitro *system in which we can control the cellular levels of YY1 protein. Briefly, human YY1 was added into the mouse cell line Neuro2A, and used as an exogenous gene, which allows for easy distinction between the exogenous and endogenous YY1. A Tet-On inducible system was employed to control the expression of the human YY1. We have successfully established several stable cell lines that can induce transcription of the human YY1 by the administration of an inducer, Doxycycline (Tet-On Advanced system) [13]. More detailed information is described in Methods.

**Figure 3:**
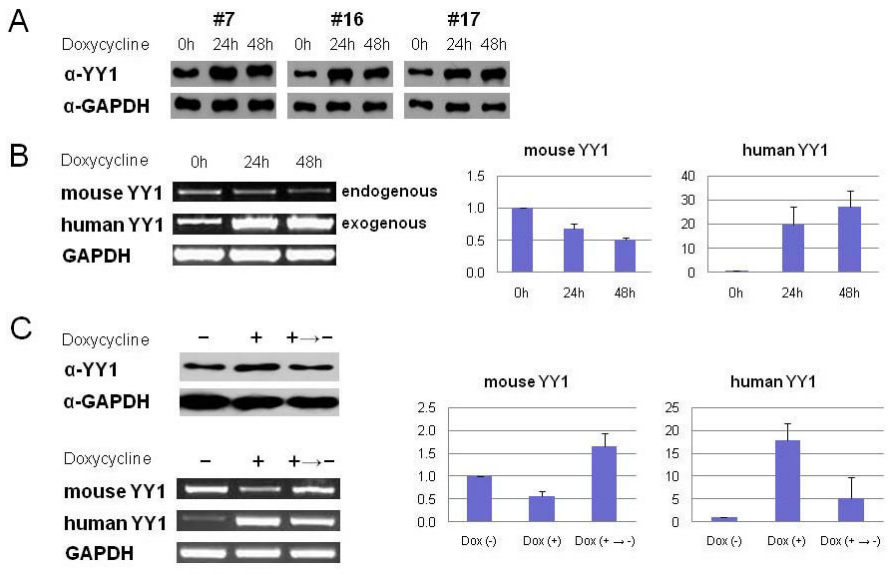
**Homeostatic responses of the YY1 locus against the fluctuating levels of YY1 protein**. A Tet-On induction system over-expressing human YY1 was established to control the cellular levels of YY1 protein in the Neuro2A cell line. Three stable YY1 inducible clones (#7, #16 and #17) were analyzed to determine the overall levels of YY1 protein at 0, 24 and 48 hours after the treatment of 1 ug/ml Dox with western blot analyses using YY1 and GAPDH antibodies (**A**). RT-PCR and quantitative RT-PCR were performed using total RNAs isolated from the same cell set as used in the above. The primer sets for RT-PCR were designed to distinguish between endogenous mouse and exogenous human YY1 (**B**). The transcriptional response from the endogenous mouse YY1 was further analyzed using a scheme which allows a cycle of up and down regulation of the human YY1 through Dox administration and withdrawal, respectively (**C**). Western blot analysis showed the concurrent up and down regulated levels of YY1 protein. Following RT-PCR and qRT-PCR also confirmed this pattern in the exogenous human YY1. As predicted, the endogenous mouse YY1 showed an opposite pattern: the down and up-regulation in response to the increased and decreased overall levels of YY1 protein.

With the three selected stable cell lines, we first performed time-course experiments (Figure 3A). All three cells showed increased levels, about 5 fold, of YY1 protein at 24 and 48 hours after the Dox administration as shown on western blot results. With the same set of cells, we further confirmed this induction at the transcription level by performing RT-PCR, in which we can differentiate the induced human YY1 from the endogenous mouse YY1 (Figure 3B). As expected, we observed a dramatic increase in the transcriptional level of human YY1 at 24 and 48 hours after the Dox administration. Although we observed low levels of the human YY1 prior to the Dox induction, this is believed to have been caused by genomic DNA contamination in the RNA samples. Interestingly, the transcriptional levels of the endogenous YY1 progressively decreased during the induction. Another independent measure using qRT-PCR further revealed that the transcriptional levels were reduced to 70% at 24 hours and to 50% levels at 48 hours, respectively. According to our survey on the transcription levels of other genes (*Peg3*, *Usp29*, *Nespas*, *Nesp*, *Xist*, and *p53*), the observed down-regulation was specific to the YY1 locus (data not shown). This indicates that the overall increased levels of YY1 protein, through the induction of the exogenous YY1, somehow resulted in reduction in the transcriptional levels of the endogenous YY1. This initial observation was further validated through the following scheme. We first increased the overall levels of YY1 protein through the induction of the human YY1, and later reversed the increased levels back to the normal levels of YY1 protein by withdrawing the inducer, Dox, from culture media. As predicted, this cycle of addition and withdrawal of the Dox treatment caused a concurrent rise and fall in the overall levels of YY1 protein. The levels of endogenous YY1 transcription changed in a manner inverse to the induced YY1 protein: decreasing with YY1 induction and increasing with removal of YY1 induction as shown in the results of qRT-PCR (Figure 3C). This inverse pattern is thought to be a counter-balancing response from the endogenous YY1 locus to maintain constant cellular levels of YY1 protein. In sum, these results confirm that the overall cellular levels of YY1 protein can affect the transcription levels of the YY1 locus.

### YY1 is autoregulated through its own binding sites

**Figure 4:**
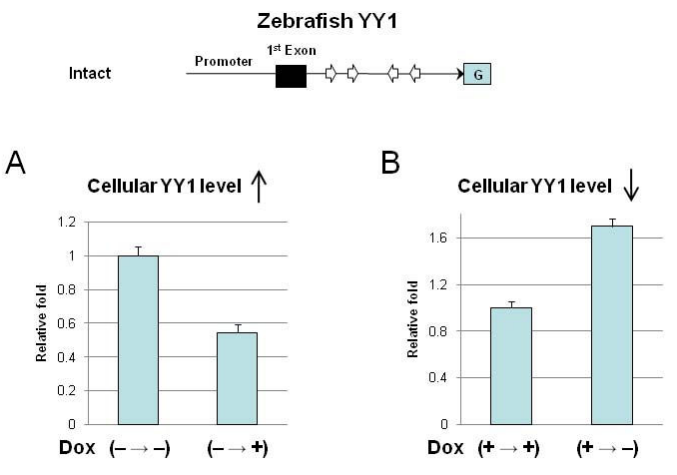
**YY1 is autoregulated through its own binding sites**. To demonstrate autoregulation through YY1's internal binding sites, we have analyzed the transcriptional activity of the zebrafish YY1 promoter with/without the YY1 binding sites at the two different cellular level of YY1 protein. These different conditions were set up using a Tet-On induction system over-expressing exogenous YY1. Before the transfection, the cell line was incubated in the absence (**A**) and presence (**B**) of the inducer, Dox. During transfection, Dox was added (**A**) and removed (**B**) to make the YY1 protein levels higher and lower, respectively, that the control cell line. Two constructs, Control and All mut, were individually transfected into the two different inducible cell lines, #7 and #16, along with the internal control luciferase vector (pGL3 control) in order to normalize β-gal activity.

The above results clearly demonstrated that the endogenous locus of YY1 has an unknown feedback regulatory mechanism that maintains homeostasis of the YY1 protein levels. We next tested if this observed regulation is indeed mediated through its own DNA-binding sites as part of the hypothesized autoregulation. For this test, we analyzed transcriptional responses from the YY1 promoter against a background of fluctuating cellular levels of YY1 protein. We first set up the two different cellular levels of YY1 protein with the following scheme (Figure 4). The cells to be transfected were first incubated in the absence (**A**) and presence (**B**) of Dox prior to transfection experiments. During the transfection, Dox was added (**A**) and removed (**B**) to create higher and lower levels of YY1 protein relative to the control cell, respectively. Then, the actual transfection into these cells used each of the following two reporters, Intact and All-mut constructs, containing the promoter region of the zebrafish YY1 with the intact and mutated YY1 binding sites, respectively (Figure 4). These individual transfections were also normalized by including the control luciferase vector (pGL3 control). In the first condition (Figure 4A), the promoter activity was measured while the cellular levels of YY1 protein were being increased by the Dox induction. As shown in Figure 4A, the Intact construct (containing the YY1 binding sites) showed a 0.5-fold decrease in the promoter activity when the cells started producing more YY1 protein. We also observed a similar down-regulation from the endogenous locus in response to increased levels of YY1 protein (Figure 3). This response, however, was not detected in the All-mut construct (containing the mutated YY1 binding sites), confirming that the observed response was mediated through the YY1 binding sites. In the second condition (Figure 4B), the promoter activity was measured while the cellular levels of YY1 protein were decreased by the Dox withdrawal. The Intact construct showed in a 1.7-fold increase in the promoter activity when the cells started producing less YY1 protein. This is also reminiscent of an observed up-regulation from the endogenous locus of YY1 in response to the decreased levels of YY1 protein. Collectively, the zebrafish YY1 promoter and the endogenous locus of YY1 responded very similarly to fluctuating levels of YY1 protein. These similar responses agree very well with the initial prediction that the cluster of YY1 binding sites most likely mediates the hypothesized autoregulation mechanism for the endogenous locus of YY1.

## Discussion

The current study reports the three unique features associated with the cluster of YY1 binding sites that is localized in the 1^st ^intron of the vertebrate YY1 locus. First, this cluster of YY1 binding sites are well conserved throughout all the vertebrates including urochordates, suggesting that this small region has been functionally selected in the past 500 to 600 million years (Figure 1). This region is probably one of the oldest *cis*-regulatory regions in the vertebrate genomes. However, similar regions are not found in the *pho *locus, the YY1 homolog in flying insects, suggesting the formation of this region after the divergence of vertebrates and invertebrates. This sudden formation of this region in the vertebrates' YY1 locus may be linked to the different expression profiles of this gene between these two groups of animals. In insects, the homeotic genes including *pho *are transiently expressed by segmentation genes during early embryonic development, and their later expressions are controlled by two groups of genes, the trithorax group (trxG) and the Polycomb group (PcG) [14,15]. Therefore, the expression pattern of *pho *may be restricted and focused in a developmental stage-specific manner. In contrast, YY1 is expressed ubiquitously, spatially and temporarily [16]. The ubiquitous expression may have allowed YY1 to be involved in a greater number of genes and pathways in the vertebrates than in the invertebrates. Consequently, the involvement in a large number of genes most likely has necessitated more sophisticated regulation mechanism(s) for the maintenance of appropriate cellular levels of YY1. Taken together, the cluster of YY1 binding sites most likely has coevolved with the ubiquitous expression pattern of YY1.

The second unusual feature is that this conserved *cis*-regulatory region has the DNA-binding sites for its own protein product, YY1. This immediately suggests a potential autoregulation mechanism for this locus. According to our cell line studies, the cellular levels of YY1 protein indeed affected the transcriptional levels of its own gene locus (Figure 3), and this control also appeared to be mediated through the cluster of YY1 binding sites (Figure 4). Besides being part of this predicted autoregulation, the cluster of YY1 binding sites is also regarded as a transcriptional activator (Figure 2) or repressor (Figure 3 and 4). Any mutations in the YY1 binding sites caused a dramatic decrease in the transcriptional activity of the YY1 promoter, indicating that YY1 is required for the transcription of its own locus. On the other hand, the same cluster of YY1 binding sites functions as a repressor in the presence of excessive cellular levels of YY1 protein. According to surveys on transcriptional regulatory networks [17], many *E coli *proteins, especially repressors, are also subject to similar autoregulation. Furthermore, the observed autoregulation is similarly 'negative' autoregulation: these repressors start repressing their own loci once their protein levels reach at their threshold levels. Despite these similarities, however, it is important to note that this cluster of YY1 binding sites has dual roles, repressor and activator. Interestingly, these roles have been detected at different cellular conditions: an activator role during the promoter assays with a normal level of YY1 protein (Figure 2) while a repressor role during YY1 induction with higher levels of YY1 protein (Figure 3 and 4). This suggests that the available cellular levels of YY1 protein may be a factor deciding which of the two roles to be exerted for a given condition.

Then, the next question is how this cluster of YY1 binding sites knows different cellular conditions and selects a role between an activator and repressor function. This question may be related to the third feature of this cluster of YY1 binding sites: multiplicity. One plausible scenario would be that the cluster of YY1 binding sites could function as a sensor recognizing the cellular levels of YY1 protein. In the case of higher-than-normal YY1 levels, all of the YY1 binding sites would be filled with the YY1 protein, subsequently block the traverse of this region by Pol II polymerase, and thus function as a repressor. In the case of lower-than-normal YY1 levels, on the other hand, only one or two YY1 binding sites would be filled, subsequently stimulating transcription by Pol II, and thus act as an activator. It will be very interesting to test this possibility in the near future, but the multiplicity and evolutionary conservation associated with the YY1 binding sites are very unusual, and clearly suggest some unknown functional roles in this regulatory region.

Despite a large number of genes that are controlled by YY1, there have not been a lot of studies on the transcriptional control of YY1 itself. Although the current study has mainly focused on the predicted autoregulation mechanism that might be mediated through the cluster of YY1 binding sites, we believe that this is not the only regulatory mechanism responsible for the YY1 transcription. Also, we cannot rule out the possibility that the identified cluster of YY1 binding sites might be designed for some other functions for the YY1 locus, such as a targeting site for epigenetic modification or a promoter for some unknown transcripts. In a similar line, it is important to emphasize the fact that the unusually high levels of YY1 protein are often observed in normal and cancer cells [3]. The up-regulated YY1 levels in normal cells may be an outcome of an independent regulatory program that has been preset in a given cell. On the other hand, the unusually high levels of YY1 protein observed in cancer cells might be an outcome of mis-regulated YY1 transcription. One intriguing possibility would be that some epigenetic changes, such as DNA methylation or histone modification, and subsequent malfunction of the cluster of YY1 binding sites might be responsible for this mis-regulation. In that regard, it will be interesting to test the epigenetic modification of this region in cancer cells. Overall, the cluster of YY1 binding sites described in the current study represents a very unique example of eukaryotic *cis*-regulatory region.

## Conclusion

The current study has identified a very unusual cluster of YY1 binding sites within its own gene locus, prompting a potential autoregulation mechanism for YY1 transcription. Consistently, the overall cellular levels of YY1 protein were shown to affect (or control) the transcription levels of the endogenous YY1 locus. Also, this control appears to be mediated through its own YY1 binding sites. This suggests that YY1 is likely self-regulated through its own binding sites.

## Methods

### Sequence alignment

The cluster of YY1 binding sites in the first intron of YY1 was discovered through the ECR (Evolutionary Conserved Region) browser [9]. This conserved region was manually aligned from genomic sequences of each genome ranging from urochordate (sea squirt) to placental mammals. A series of database searches were conducted using the BLAST program  to obtain the genomic sequences of the YY1 locus from several species. The aligned sequences are as follows: sea urchin (Scaffold81718: 20284–20353), sea squirt (ENSCSAVG00000003120: 624148–624214), amphioxus (JGI scaffold_8: 4922034–4922089), zebrafish_1 (NC_007128: 40211168–40211267), zebrafish_2 (NW_001877837: 59200–59296), fugu_1 (chrUn: 161085228–161085087), fugu_2 (chrUn: 51268538–51268643), human (NC_000014: 99776064–99776201), mouse (AC_000034: 110030364–110030467), cow (NC_007319: 58875242–58875872), dog (NW_876327: 66420933–66421058), opossum (ENSMODG00000013124: 318625672–318625815), and frog (JGI4.1:scaffold_222: 1017795–1034375).

### ChIP assay

ChIPs were performed according to the protocol provided by Upstate Biotechnology with some modification as described previously [18]. Briefly, the mouse brain tissues (0.1 g per tissue) were homogenized in 10 ml PBS for ChIP assay. The samples were treated with formaldehyde to a final concentration of 1% and incubated at 37°C for 10 min. Treated samples were sheared by sonication to derive DNA fragments averaging 500 bp in length. Sheared chromatin was immunoprecipitated with anti-YY1 antibody (sc-1703; Santa Cruz Biotechnology). Precipitated DNA and protein complexes were reverse crosslinked and purified through phenol/chloroform extraction. Purified DNA was used as templates for PCR amplification. Three primer sets were used: ChmYYup1(+), 5'-GGCACTTTTGTCACTGTTGCACCGCG-3' and ChmYYup2(-), 5'-CAACTCCTCAACCCCGAGCCCAGATCTC-3'; ChmYYb3(+), 5'-GGGAGCAGAAGCAGGTGCAGATCAAG-3' and ChmYYb4(-), 5'-CTCAACCGGCCCCGCCGCACGTCCGTTG-3'; ChmYYdw5(+), 5'-CTGCACGGTAGGTTATCAGGAGCTGTATG-3' and ChmYYdw6(-), 5'-CGATTCATCAACACCACACTTGACGAAG-3'. PCR reactions with an inclusion of 1% DMSO were carried out for 35 cycles (94°C, 30 s; 64°C, 30 s; 72°C, 30 s) using the Maxime PCR premix kit (Intron Biotech). The resulting PCR products were analyzed by running on 1.5% agarose gel containing ethidium bromide. The animal experiment was performed in conditions approved by the Louisiana State University Institutional Animal Care and Use Committee (IACUC), protocol #07–051.

### Promoter assay

We used one β-Geo vector that has been modified from pGT1.8iresβgeo [19]. We have transferred a *Bam*HI partial digested fragment containing the IRES-LacZ-Neo cassette into the *Bam*HI site of pBluescript II SK (+). All constructs derived from zebrafish YY1 (BX546455; 5043–6110) were cloned into the *Not*I site of this IRES-β-Geo vector. Several mutations on 4 YY1 binding sites were performed by PCR-based mutagenesis with modified oligonucleotides. Neuro2A and NIH3T3 cells were maintained in MEM and DMEM medium with 10% fetal bovine serum and 1% antibiotic-antimycotic (GibcoBRL), respectively. 2×10^5 ^cells were plated in each well of a six-well plate. On the next day, cells were co-transfected with GeneJuice transfection reagent according to the manufacturer's protocol (Novagen). Two days after transfection, the cells were harvested, washed with PBS, and treated with 100 μl of lysis buffer (0.25 M Tris-HCl, pH 7.8 + 0.1% NP40) for 30 min at 4°C and cellular debris was removed by centrifugation for 10 min. For the β-galactosidase assay, 30 μl of cell lysate was mixed with the same volume of 2× β-galactosidase assay buffer (Promega) in a 96-well flat bottom clear plate. The plate was incubated at 37°C, monitored visually and terminated with 90 μl of 1 M sodium carbonate. The absorbance was measured at 405 nm with Wallac 1420 multilabel counter VICTOR^3 ^(PerkinElmer). For the luciferase assay, 20 μl of cell lysate was combined with 100 μl of Luciferase Assay Reagent (Promega) in a 96-well flat bottom white plate (Corning). Luminescence was measured with Wallac 1420 multilabel counter VICTOR^3^. To control for transfection efficiency in each well, β-galactosidase activity was normalized to luciferase activity.

### YY1 inducible cell line

pTet-On Advanced, pTRE-tight vectors and HeLa Tet-On cell line were purchased from Clontech. The cDNA fragments encoding human and mouse YY1 (GenBank accession No. NM_003403 and NM_009537) were inserted into BamHI-NheI sites and BamHI-NotI sites of the pTRE-tight vector, respectively. Neuro2A Tet-On cell line was established by stable transfection of the pTet-On Advanced vector under the presence of 500 ug/ml G418. The human YY1 and mouse YY1 cloned pTRE-tight vectors were cotransfected into Neuro2A and HeLa Tet-On cell lines with the linearized Hyg^r ^gene at a 10:1 molar ratio, respectively. Eight single colonies in the Neuro2A Tet-On cell line only were isolated in the presence of 250 ug/ml G418 and 500 ug/ml hygromycin B. The clones were analyzed to determine the cellular levels of YY1 protein by western blot analysis after doxycycline treatment.

### Western blot analysis

For our western blot analysis, the cells were lysed after different incubation times following doxycycline treatment for 30 min at 4°C using lysis buffer (0.25 M Tris-HCl, pH 7.8, plus 0.1% NP-40). Cellular debris was removed by centrifugation for 10 min. Protein concentrations were determined by the Bradford assay kit (Pierce). Thirty micrograms of lysate was separated on 10% SDS-PAGE gels and transferred to PVDF membrane (Hybond-P; Amersham) using a Mini Trans-Blot transfer cell (Bio-Rad). Membranes were blocked for 1 h in Tris-buffered saline containing 5% skim milk and 0.05% Tween 100 and incubated at 4°C overnight with anti-YY1 (sc-1703; Santa Cruz Biotechnology) or anti-GAPDH (MAB374; Chemicon) antibodies. These blots were incubated for an additional 1 h with the secondary antibody linked to horseradish peroxidase (Sigma). The blots were developed using a Western blot detection system according to the manufacturer's protocol (Intron Biotech).

### RT-PCR and qRT-PCR

Total RNAs were first purified from transfected cells using Trizol as described by the manufacturer (Invitrogen); second, first-strand cDNA was reverse transcribed using the SuperScript First-Strand Synthesis System (Invitrogen); and finally PCR amplifications were performed using the following primer sets: human YY1 (hYY1F-2, 5'-GACCTCTCAGATCCCAAA-3' and hYY1R-1, 5'-TTGTTTTTGGCCTTAGCA-3'), mouse YY1 (mYY1F-2, 5'-GACCTCTCAGACCCTAAG-3' and mYY1R-1, 5'-TTGTTTTTGGCTTTAGCG-3'), and mouse GAPDH (GAPDH-RT-F, 5'-ATGACATCAAGAAGGTGGTG-3' and GAPDH-RT-R, 5'-CATACCAGGAAATGAGCTTG-3'). Also, quantitative real-time PCR was performed with iQ SYBR Green Supermix (Bio-Rad) using the icycler iQ multicolor real-time detection system (Bio-Rad). All qPCRs were carried out for 40 cycles under standard PCR conditions. We analyzed the results of quantitative real-time PCR based on the ΔΔCt method [20].

## Authors' contributions

JDK analyzed sequences and constructed all the vectors for the promoter assays, and also performed the ChIP experiment and wrote the paper. SY established the YY1 inducible cell lines and performed RT-PCR, western blot and the promoter assays. JK provided the original concept of the study, supervised the study, and contributed to writing the paper. All authors read and approved the final manuscript.
